# Case Report: Thoracic Aortic Dissection in a Previously Healthy Male with an Unusual Inciting Factor

**DOI:** 10.21980/J8G92S

**Published:** 2021-07-15

**Authors:** Peter L Vuongv, Edward J Durant, Christopher B Branham

**Affiliations:** * Kaiser Permanente Central Valley, Department of Emergency Medicine, Modesto, CA

## Abstract

Thoracic aortic dissection is a life-threatening emergency that can be easily overlooked in the absence of commonly associated risk factors, such as hypertension, connective tissue disease, cocaine use, and older age. The classic presenting symptom of abrupt-onset, severe “tearing” or “ripping” chest pain may be an unreliable finding. We present the case of a 30-year-old previously healthy male with no known risk factors who turned around to sanitize after having a bowel movement when he began feeling tight, severe back pain. After ruling out common etiologies of acute back pain with a negative computed tomography (CT) scan, point-of-care ultrasonography was performed which revealed a crescent-shaped flap within the abdominal aorta. Follow-up computed tomography angiogram (CTA) confirmed an extensive Stanford type A aortic dissection. Remarkably, the patient remained hemodynamically stable throughout his emergency department (ED) visit without developing any new pulse deficits or heart murmurs. It is a good reminder to maintain a high index of suspicion for aortic dissection in patients presenting with acute back pain without any classic risk factors. We also review the most recent literature regarding aortic dissection in young adults and ultrasonography for aiding diagnosis in the ED.

**Topics:**

Thoracic aortic dissection, aortic dissection risk factors, CT scan, point-of-care ultrasonography.

**Figure f1-jetem-6-3-v23:**
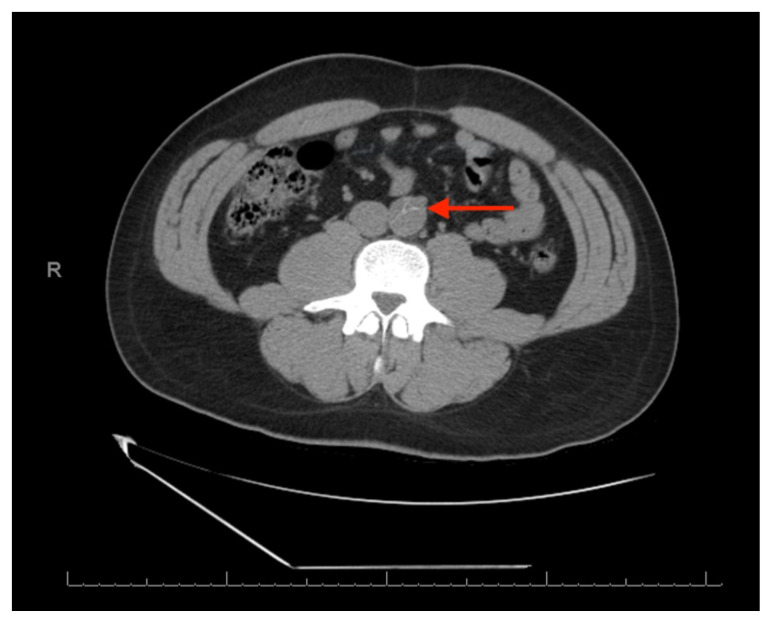


**Figure f2-jetem-6-3-v23:**
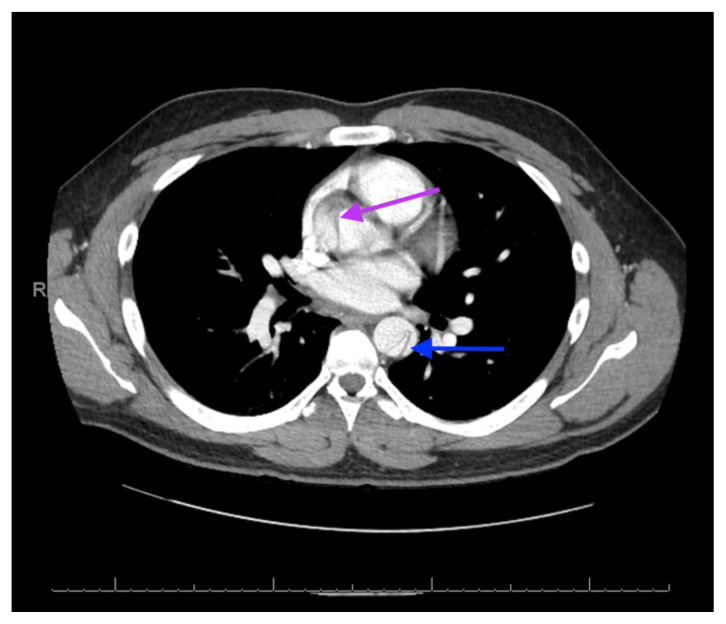


## Brief introduction

[Fig f1-jetem-6-3-v23][Fig f2-jetem-6-3-v23]Acute aortic dissection classically presents as severe chest or back pain and is commonly associated with certain predisposing factors such as older age and hypertension.^1^ Although there are reports in the literature of aortic dissection in young patients as well, these are typically associated with underlying connective tissue disease or a history of aortic surgery.^2^ We present an unusual case of thoracic aortic dissection in a patient without any of the classic risk factors or precipitating events, such as trauma, but rather caused by a seemingly benign event at home.

## Presenting concerns and clinical findings

A previously healthy 30-year-old man presented to the ED with four hours of acute low back pain. The pain was described as tight, constant, and severe without radiation and began after turning his upper body to sanitize after a bowel movement at home. Associated symptoms included lightheadedness, nausea, and vomiting. He denied dyspnea, chest pain, abdominal pain, hematochezia, hematuria, dysuria, or recent trauma. There was no popping or tearing sensation felt at the time of onset. He had tried ibuprofen along with ice and heat which provided little symptomatic relief. He was a lifetime nonsmoker and denied alcohol or illicit substance use, including cocaine. Medical, surgical, and family histories were unremarkable.

Upon arrival to the ED, the patient had a blood pressure of 120/52 mmHg, temperature of 36.6°C, heart rate of 64 beats/min, respiratory rate of 17 breaths/min, and 98% oxygen saturation on room air. His BMI was 31.5 kg/m^2^. On examination, the patient appeared distressed. Cardiovascular examination revealed normal heart sounds, no audible murmurs and strong, equal pulses in all extremities. His abdomen was noted to be soft without guarding or masses. He was noted to have point tenderness at the thoracolumbar junction in addition to mild costovertebral tenderness on the left side. He ambulated well and had no focal neurological deficits or signs of weakness.

## Significant findings

The patient’s labs were notable for a white blood cell count of 28,900/mm^3^, potassium of 3.0 mEq/L, and lactate of 6.2 mEq/L. Urinalysis results, erythrocyte sedimentation rate, and C-reactive protein levels were within normal limits. A noncontrast computed tomography (CT) scan was negative for a suspected ureteral stone. However, there were aortic calcifications visualized at the infrarenal level that were notable given the patient’s age (red arrow). Given this finding in conjunction with the patient’s symptoms, bedside transabdominal ultrasonography was performed which revealed an intraluminal echogenic flap within the aorta near the common iliac arteries. CT angiography (CTA) with delayed contrast protocol revealed an extensive Stanford type A aortic dissection with involvement of the aortic root (purple arrow), brachiocephalic trunk, ostia of the left subclavian artery, descending aorta (blue arrow), bilateral common iliac arteries, and left internal iliac artery.

## Patient course

Given the CTA findings, a vascular surgery consult was obtained which recommended immediate transfer to the closest facility with cardiothoracic surgery capabilities given the finding of elevated lactate levels and concern of mesenteric ischemia. An intravenous esmolol drip was initiated. The patient remained hemodynamically stable throughout his entire ED visit. There were no new findings of pulse deficits, heart murmurs, or tachycardia. Repeat laboratory results taken 4 hours into his ED stay revealed no significant changes. He was transferred in stable condition to another hospital where he underwent successful endovascular repair and had an otherwise uneventful hospital course. He was discharged home seven days later.

## Discussion

Thoracic aortic dissection is rare and almost invariably fatal when missed, so it is imperative for the clinician to maintain a high degree of suspicion because it can present atypically. While its incidence is estimated to be only 3 to 4 cases per 100,000 persons per year, the rate of misdiagnosis can be as high as 39%, most commonly mistaken for acute coronary syndrome.^3^,^4^ The most common presenting symptom is the abrupt onset of severe chest pain (85%), followed by back pain (53%).^5^ Back pain is more often associated with type B dissections than type A dissections, while abdominal pain was reported in less than a third of patients experiencing either kind of dissection.^6^,^7^ Other common symptoms include murmur of aortic insufficiency, pulse deficits, and signs of end-organ ischemia such as stroke, paraplegia, renal failure, and abdominal pain.^7^,^8^

Risk factors that predispose adults to aortic wall damage and lead to dissection include chronic hypertension, smoking, dyslipidemia, trauma, iatrogenic procedures involving the aorta, connective tissue disorders (eg, Marfan syndrome, Loey-Dietz syndrome, Ehlers-Danlos syndrome), and cocaine abuse.^5^ Age and male sex are also correlative, with mean age of occurrence at 61.5 years and an approximately 2:1 male-to-female ratio.^3^

With regard to clinical presentation in younger patients, an analysis of 68 patients under the age of 40 from the International Registry of Acute Aortic Dissection found that they were half as likely to have a prior history of hypertensi on at initial presentation than older patients.^2^ However, they were much more likely to have Marfan syndrome, bicuspid aortic valve, or previous aortic surgery. In another case series that examined thirteen patients under the age of 25, nine patients had either congenital heart defects or Marfan syndrome while one patient had no identifiable risk factors.^9^ This data suggests that young patients newly diagnosed with thoracic aortic dissection should also be evaluated for genetic or structural causes in order to tailor treatments accordingly and to screen family members who may be at risk.^10^

Our patient presented with severe back pain but otherwise lacked many of the well-reported features of acute aortic dissection. Careful history-taking and physical examination were critical in guiding the next diagnostic step, even if to rule out potential etiologies. Initial considerations were given to musculoskeletal and urologic etiologies, for which a non-contrast CT scan was performed and revealed aortic densities but was not interpreted as an aortic dissection. Given that a specific source of pain was not easily identified at first, along with the benign nature of the provoking factor (turning around to wipe following a bowel movement) in an otherwise healthy young male, this patient could have been easily discharged home had aortic dissection been overlooked. In fact, one retrospective study that examined patients with missed aortic dissection in the ED found that those initially presenting with back pain were most likely to be misdiagnosed with urolithiasis.^11^

While bedside transabdominal ultrasonography is not typically utilized in the workup of aortic dissection, its use was paramount to leading to a correct diagnosis after a falsely reassuring non-contrast CT read. This permitted the emergency physician the ability to visualize an echogenic flap within the abdominal aorta which prompted the decision to follow-up with a CTA, the preferred imaging modality for accurate diagnosis of aortic dissection. This approach is supported by a study that found that emergency physicians who included focused bedside ultrasonography achieved a 0% misdiagnosis rate (versus 44% in those who did not) while also reducing time to diagnosis.^12^ In our case, nonspecific findings on initial CT along with a high index of suspicion supported the decision-making. Nonetheless, bedside ultrasonography should be considered as a helpful tool in speeding clinical investigation of suspected aortic dissection.

## Supplementary Information








