# Supplementary figures and images for: Case Report: Thoracic Aortic Dissection in a Previously Healthy Male with an Unusual Inciting Factor
Source: J Educ Teach Emerg Med. 2021 Jul 15;6(3):V23–6. doi: 10.21980/J8G92S (PMC10332694; doi:10.21980/J8G92S)

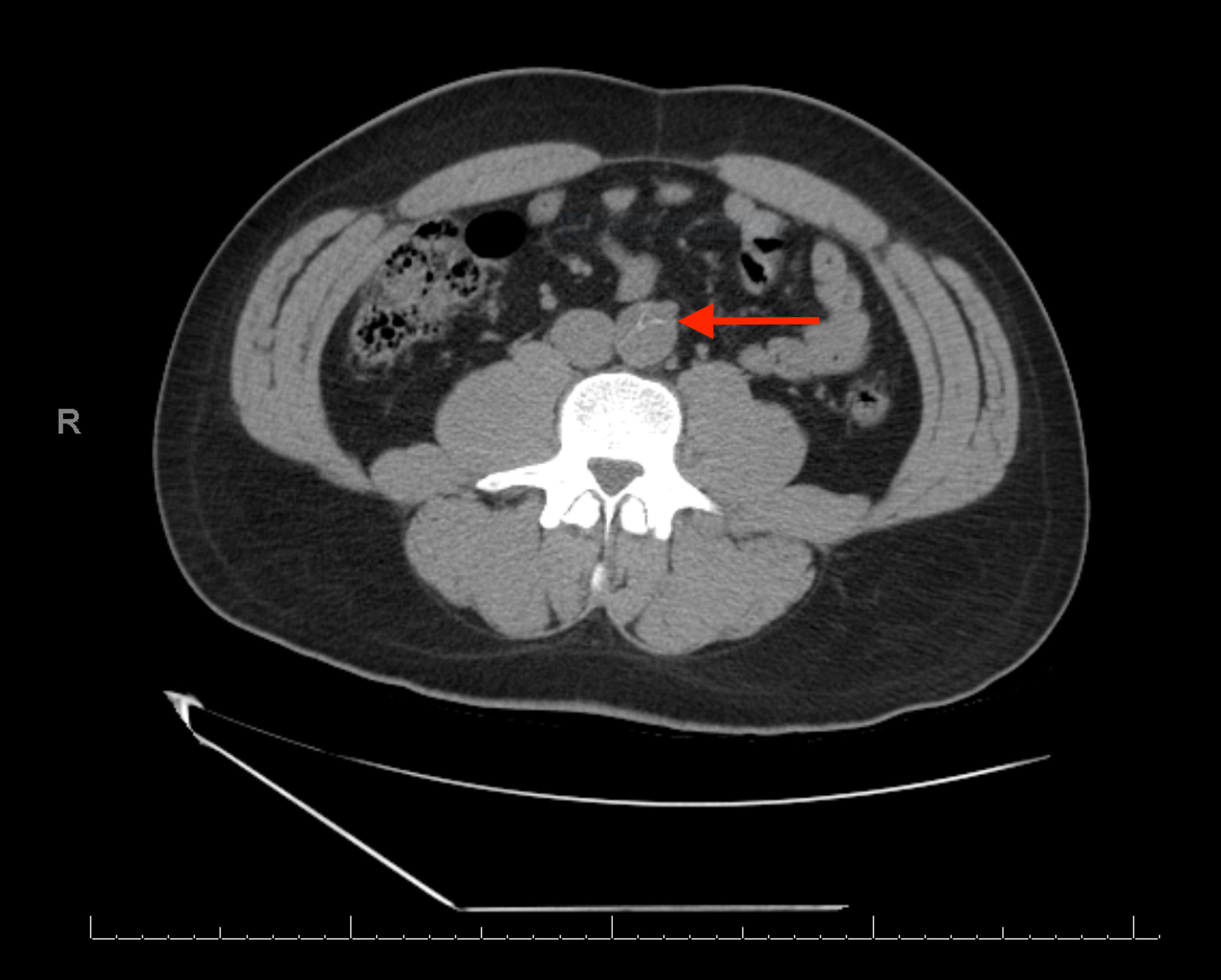

Supplement: Supplementary file 1 [file jetem-6-3-v23-supp1.jpeg]

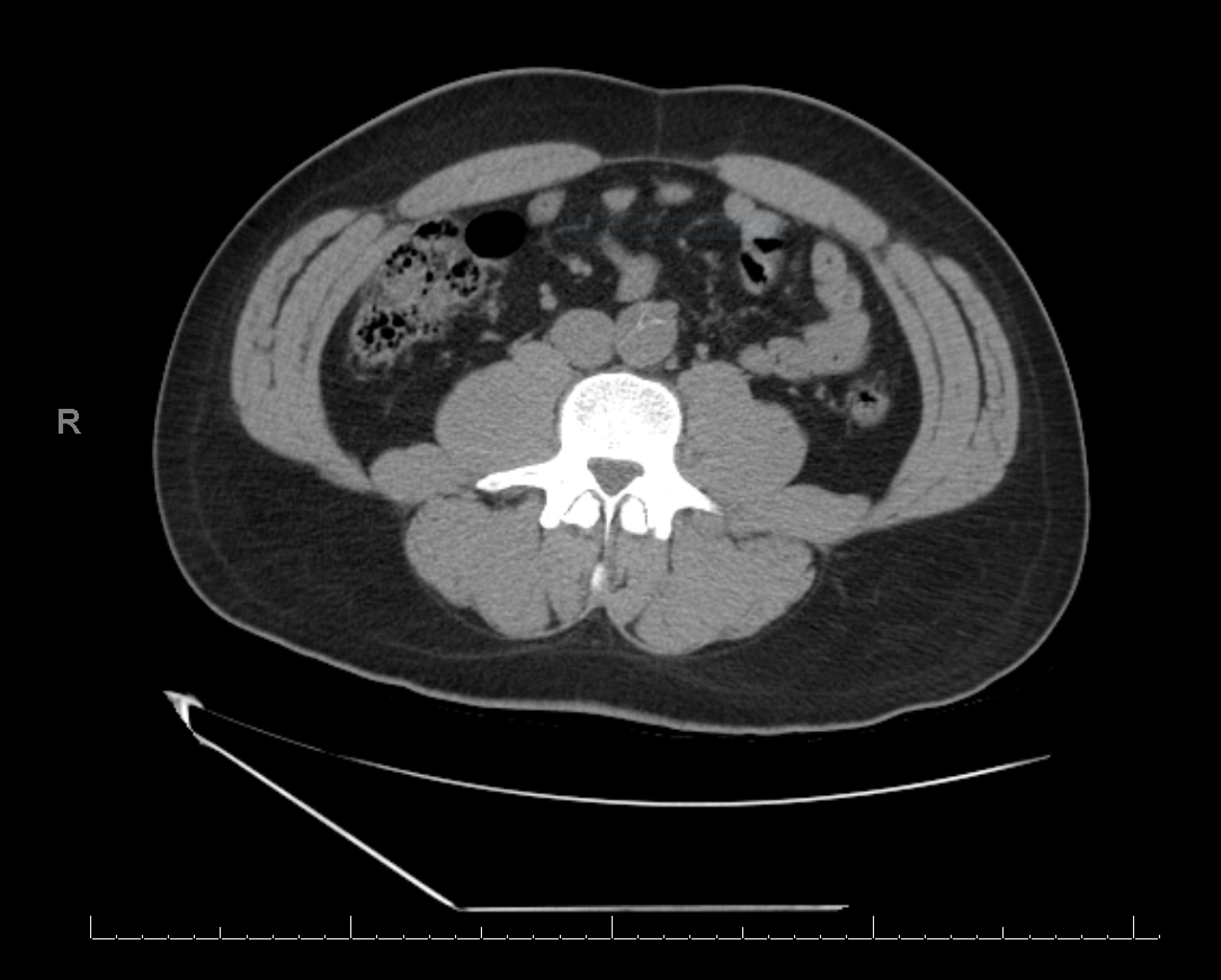

Supplement: Supplementary file 2 [file jetem-6-3-v23-supp2.jpeg]

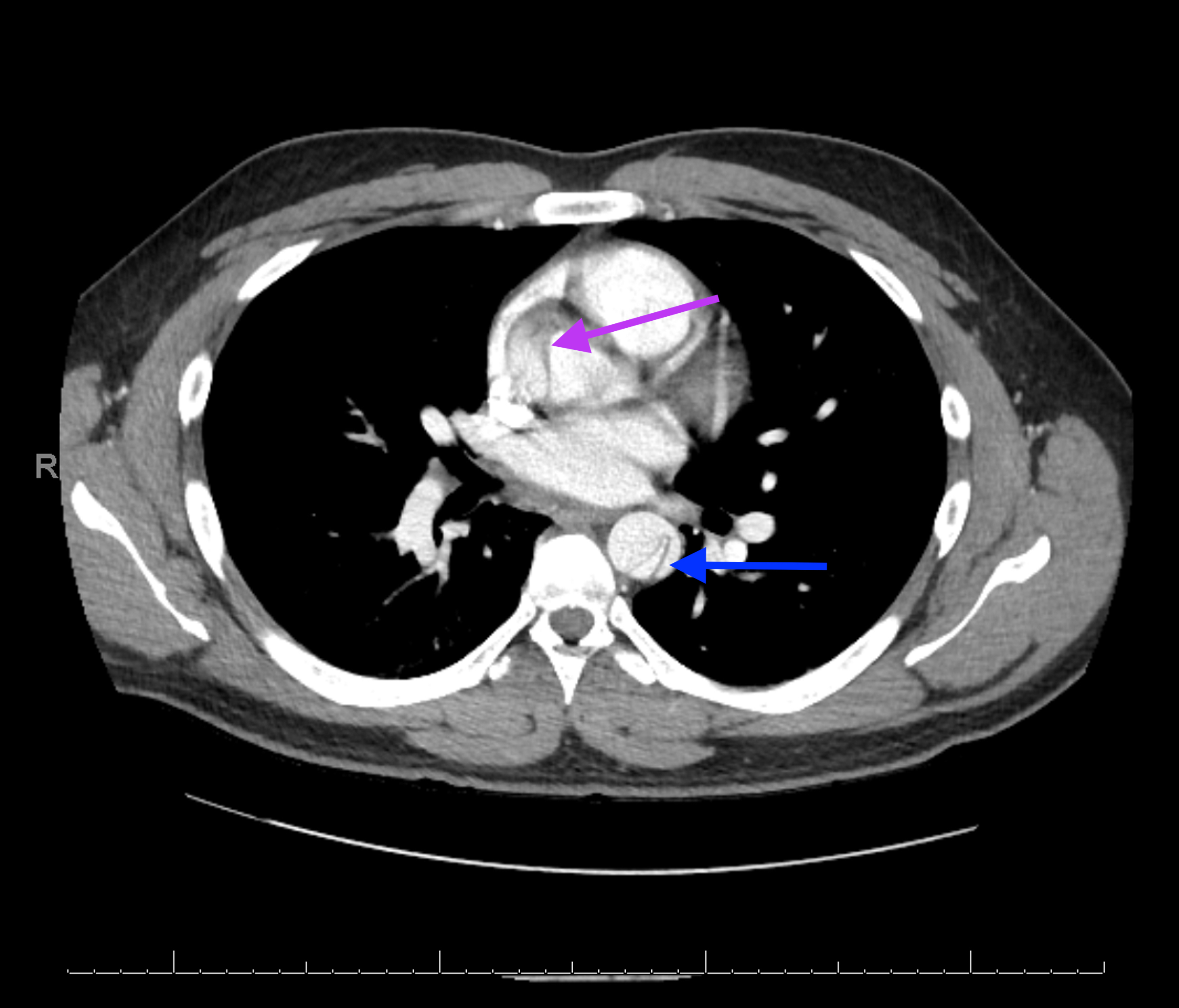

Supplement: Supplementary file 3 [file jetem-6-3-v23-supp3.jpeg]

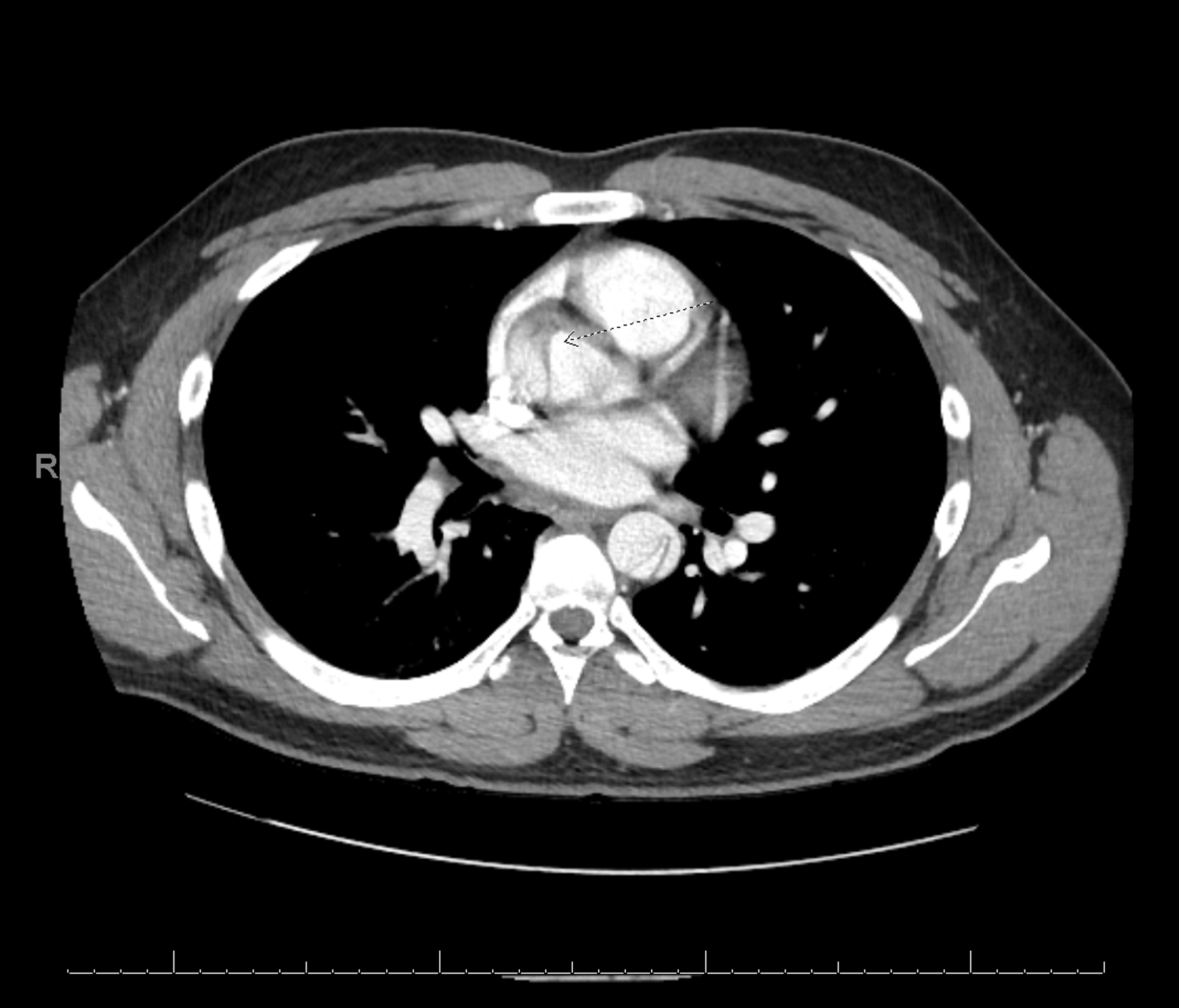

Supplement: Supplementary file 4 [file jetem-6-3-v23-supp4.jpeg]
